# Comparison of clinical parameters of peri-implantitis and parameters related to tissue macrophage sensitization on TiO_2_

**DOI:** 10.1007/s00784-024-05880-3

**Published:** 2024-08-25

**Authors:** Philipp Sahrmann, Jens Tartsch, Patrick R. Schmidlin

**Affiliations:** 1 Department of Periodontology, Endodontology and Cariology, University Centre for Dental Medicine, University of Basel, Basel, Switzerland; 2 Private Practice, Kilchberg, Switzerland; 3 Clinic of Conservative and Preventive Dentistry, Center of Dental Medicine, University of Zurich, Zurich, Switzerland

**Keywords:** Peri-implantitis, Macrophage sensitization, Titanium dioxide, Risk factors

## Abstract

**Objective:**

Dental implants show impressive survival and like rates, but peri-implantitis is a frequent inflammatory disease which affects the implant-surrounding tissues. While biofilms on the implant surface is considered its etiologic reason, several risk factors determine the pace of progression of peri-implant bone loss. Some risk factors are generally accepted while others are still unconfirmed and a matter of ongoing discussion. Among the latter, tissue macrophage sensitization on TiO_2_ has gained scientific interest in recent years. The aim of the present case-control study was to test for potential associations between clinically manifest peri-implantitis and MS related parameters.

**Materials and methods:**

In patients with implants affected by peri-implantitis in the test group and healthy implants in the control group clinical parameters (peri-implant pocket depths (PPD) and bleeding on probing (BOP) were measured. Samples of aMMP-8 were taken from the entrance of the peri-implant sulcus and bacterial samples were collected from the sulcus. Blood samples were obtained from the basilic vein to assess MA-related laboratory parameters. Potential correlations between clinical and laboratory parameters were tested by multiple regression (*p* < 0.05).

**Results:**

No statistically significant correlations were found between clinical or bacteriological findings and laboratory parameters were found.

**Conclusions:**

Based on the findings of this study elevated MA-related laboratory parameters do not appear to be linked to peri-implantitis.

**Clinical relevance:**

Sensitization on TiO_2_ is not associated with clinical symptoms of peri-implantitis.

## Introduction

Dental implants have demonstrated impressive survival and success rates [1–5] However, they are not without flaws, and various complications are frequently reported in clinical studies [6, 7]. Of these, complications of biologic nature are not only the most frequent ones but also often most difficult to manage [8, 9]. Within the biologic complications, peri-implant inflammations affecting the soft and hard tissues surrounding the implant are the most prevalent and potentially impactful, leading to implant failure and loss [5]. The minor or initial form, peri-implant mucositis, only affects the marginal host tissues, does not constitute a clinical problem for implant success and survival per se but is of utmost importance as an easy-to-treat precursor of peri-implantitis. The latter however affects all peri-implant tissues and results in the - largely irreversible - downgrowth of the bony implant attachment [9, 10]. Today peri-implantitis with a weighted mean prevalence of 22% [11] is a frequent and clinically most relevant challenge in dental practice [12].

The primary etiologic reason for these inflammatory diseases is biofilm which colonizes the implant surfaces and triggers the response of the host’s immune defense mechanisms [13, 14]. The dynamics of the disease however, i.e., the progressive destruction of the bony implant attachment, are determined by a broad variety of risk factors. History of periodontitis, smoking and diabetes mellitus are well-established and scientifically confirmed examples for such risk factors, while several further conditions have been proposed but are still lacking evidence so far [15, 16]. Among these proposed risk factors the implant material itself has gained scientific attention during the last decades [17–19]. While metal alloys with a predominant proportion of titanium have been proven to be a most reliable material for both, safe osteointegration and long-term stability of dental implants, titanium particles have been shown to corrode from the surface and to disintegrate from fixture and abutment due to micromovements between fixture and abutment [20], and then spread into the peri-implant host tissues. There, they might challenge the host’s immune response. Tissue macrophages incorporate such microparticles and - as a consequence to impossible degradation of such particles - trigger an accelerated inflammatory cascade [18]. The intensity of this cascade however seems to underly strong inter-individual differences. In part, these differences are explained by the genetic configuration of areas encoding for the expression of the cytokines tnf-α and IL-1α and IL-1ß. [21] Dependent on an either mono or bi-allelic polymorphisms in these areas, the extent of the immunologic response has been reported to vary strongly in in-vitro stimulations of blood-born macrophages with TiO_2_. [22]. In vivo, enhanced macrophage stimulation has been reported to be associated to the loss of Brånemark implants and was proposed as independent risk factor for implant failure [21]. In a case-control study on healthy implants and implants with peri-implantitis, significant differences were found in the carriage rate of one allele of a IL-1RN encoding gene sections [23]. Titanium is no longer the only implant material available and alternative materials have gained clinical relevance. Among these, ceramic and especially ZrO_2_, which is meant not to induce similar immunologic reactions, have become clinically interesting and widely used. Accordingly, and with the aim to assess the prognosis of dental implants in the individual, a macrophage stimulation test on TiO_2_ has been proclaimed an important measure before implant therapy. Depending on the result the choice of ceramic as an alternative has been suggested if the patient shows respective polymorphisms and an accelerated immunologic response [24, 25].

However, the issue remains controversial based on recent reviews, and whether polymorphisms in the encoding sections for IL-1ß and tnf-α are in fact having an effect on or whether they are associated with peri-implant inflammations [16, 26–28] is still matter of ongoing discussion. These reviews do accord, however, on the fact that further clinical data is needed to understand the potential impact of such polymorphisms.

Therefore, it was the aim of the present case control study to assess a potential correlation of the of peri-implant health and TiO_2_-sensitisation-related parameters.

## Materials and methods

The hypothesis of the present study was, that patients with clinical symptoms of peri-implantitis display a higher degree of gene section polymorphisms encoding for the pivotal cytokines tnf-α and IL and related cytokine reactions on TiO_2_. Prior to study start an ethics approval was obtained by the Ethical Committee of the Canton of Zurich (KEK-ZH-Nr. 2014 − 0133).

Patients presenting themselves with dental implants in the Clinic of Conservative and Preventive Dentistry at the Center of Dental Medicine of the University of Zurich were asked to participate. They were informed about the aim of the study and the respective study-specific measures, and eventually gave their written consent to participate.

Patient participation was strictly voluntary.

### Patient screening

The study inclusion criteria comprised at least one dental implant which had been loaded for at least 24 months. Patient had to be older than 18y of age and able to understand the patient information (written in German). Exclusion criteria consisted of intake of systemic antibiotics or any peri-implantitis treatment in the previous 6 months. Patients with anticoagulation medication other than 100 mg of acetylsalicylic acid per day were excluded in order not to face problems after blood sampling were excluded, and patients with systemic medication for immune suppression since their immunologic response might not be representative. Heavy smokers (> 10 cigarettes/day) and patients with suspect for implant fracture were excluded likewise.

In patients presenting with implants, examinations were conducted by specialists in periodontitis and sound clinical expertise in peri-implantitis (PRS, PSA), both of which were trained to apply a probing force of 0.2 N. Probing depths were measured around the implant and at the neighboring teeth, and pus secretion and bleeding on probing was recorded.

Additionally, a radiograph of the implant was taken in right-angle technique and the marginal bone level around the implants was assessed.

Peri-implantitis was diagnosed when peri-implant pocket depths exceeded 4 mm accompanied by bleeding on probing and marginal bone loss around the implants as compared to either previous radiographs or in terms of localized vertical bone defects or deviations from the ideal bone level at the implant’s shoulder. Based on the diagnosis, patients were allocated to either the test group with peri-implantitis, or the control group if they presented with healthy peri-implant tissues.

### Study-related treatment

In the first study-related appointment, the implant was isolated with cotton rolls and then cleaned and dried supra-mucosally with cotton pellets. In order to assess the degree of inflammation in the peri-implant tissues a sample of sulcus fluid for aMMP-8 assessment was taken using respective paper strips which were provided by the laboratory (IMD Labor Berlin, Germany). Paper stripes were placed in the entrance of the sulcus for 30s and then removed for further analysis. To better characterize the features of peri-implant biofilms, paper tips were inserted to the peri-implant sulcus floor for bacterial analysis and removed after 10 s. Both samples were stored in separate tubes, which were labelled with the study-specific identification number. The number of bleeding sites after probing and the number of pocket depths exceeding 4 mm at the implant and the neighboring teeth were recorded. Three blood samples were drawn from the medial cephalic or cubital vein for the analysis of IgA, macrophage stimulation test on TiO_2_ (tnf-α and IL-ß) and the analysis of the genetic cytokine profile. Therefore, a tourniquet was applied to the upper arm and the extraction site was disinfected with Kodan forte^®^ (Schülke & Meyer GmbH, Norderstedt, Germany). The vein was punctured once only and three vacuette sampling tubes for the different laboratory assessments were drawn. Immediately after sampling, the needle was removed from the vein and the extraction site was compressed gently for 3 min.

Immediately after drawing the samples collection tubes were gently agitated and then labelled with the respective ID. Before shipping, samples were kept in a fridge at 7° until the courier collected the samples. Delivery to the 850 km distant laboratory (IMD Labor Berlin, Germany) was performed by an overnight courier in refrigerated lorries. The samples were processed there the following day.

In the laboratory, the gene sections encoding for IL-1ß and tnf-α were analyzed for bi-allelic polymorphisms at position + 3954 (*TaqI* restriction fragment length polymorphism) within exon 5 within the promoter region of the IL-1ß since these areas have been reported to be associated with an enhanced inflammatory response [29, 30]. The penta-allelic variable number of the tandem repeat polymorphism within the second intron of the *IL-1RN* gene and at position − 889 in the promoter region of the IL-1 α gene were also analyzed [31, 32]. Microbiologic assessment was performed using RNA-based amplification of defining specific gene sections by polymerase chain reaction (PCR). The specific analysis comprised the detection of the species *Aggregatibacter actinomycetemcomitans*,* Porphyromonas gingivalis*,* Tannerella forsythia*,* Treponema denticola*,* Prevotella intermedia*,* Parvimonas micra*,* Fusobacterium spp.*,* Campylobacter rectus/showa*,* Eubacterium nodatum*,* Eikenella corrodens* and *Capnocytophaga gingivalis/ochracea.* The laboratory’s detection report provided a semi-quantitative assessment categorized as “in the normal range” (considered as 0), or “low”, “moderate”, and “strong” elevations of the respective numbers (expressed as 0.33, 0.67 and 1.00, respectively, for further statistical analysis).

Levels of aMMP-8 were determined using a commercial laboratory-based enzyme-linked immunosorbent assay (DentoElisa aMMP-8, dentodiagnostics GmbH, Solingen, Germany). IgA-levels were determined using an immune turbidity test (Alinity, Abbott, Abbott Park, Illinois, USA). Mannose binding lectin assessment was tested performed using an immunometric ELISA test read at 450 nm (MBL Oligomer ELISA Kit, Bioporto Diagnostics, Hellerup, Denmark).

Individually adapted peri-implant therapy for patients in the test group was performed subsequently after the study-specific sampling.

### Statistical methods

A sample size calculation was performed prior to the study to estimate the necessary number of patients to test the study hypothesis. With an anticipated enhancement of 33% for the macrophage-secreted cytokines in the test group, as ample size of 19 for each, the test and control groups, was calculated to achieve a power of 80% and an alpha error of 5%.

Implants were defined as the statistical unit for data analysis. Due to a relatively small sample size, differences between groups were tested using the Mann-Whitney test for unpaired data, and continuous parameters such as tnf-α and IL-ß, while Pearson’s Chi-square test was used for ordinal variables such as presence of peri-implantitis or results of the molecular diagnostics.

Analyses were conducted to test a possible correlation between enhanced sensitization to TiO_2_ and the presence of the diagnosis of peri-implantitis. Odds ratios were calculated for the presence of peri-implantitis between both groups. The level of significance was set at 0.05. Statistical analysis was performed using the software IBM SPSS Statistics for Mac (Version 27, NY, USA).

## Results

Between March 2014 and October 2021, a total of 49 patients were included in the study. Due to logistic reasons, one blood sample could not be analyzed, resulting in 24 patients in both the test and the control groups. There were no significant differences in gender, age distribution and smoking status between the two groups (Table 1). In terms of clinical parameters, the test group showed a significantly higher number of sites with probing depths exceeding 4 mm of probing depth and bleeding-on-probing as compared to the control group (*p* < 0.001). Additionally, several bacterial strains (*P.gingivalis*,* T. forsythensis*,* T. denticola* and *P.micros*) were found significantly more frequently in test patients than in controls (16/7, 23/6, 16/5 and 17/9, respectively) (Table 2). The concentrations of IgA, MBL and several cytokines (tnf-α, IL-1a and IL-ß a) are provided in Table 1.

**Table 1:** Patient characteristics, clinical findings and laboratory parameters in the different groups

Parameter	Test	Control	*p*-value
**Sex** [n] *male/female*	10/14	14/10	0.257 ^a^
**Age** [y] *median (iqr)*	60.5 (12)	58.9 (20)	0.101 ^b^
**PPD > 4 mm** [n] *median (iqr)*	4.0 (0)	0 (0)	**< 0.001** ^b^
**IgA** [mg/dL] *median (iqr)*	218 (174)	198 (100)	0.718 ^b^
**MBL** [ng/mL] *median (iqr)*	682 (2303)	459 (1551)	0.217 ^b^
**aMMP-8** [ng/mL] *median (iqr)*	28.2 (60.7)	34 (67.7)	0.113 ^b^
**tnf-α** [pg/mL] *median (iqr)*	25.4 (37.8)	19.2 (37.1)	0.322 ^b^
**IL-1ß** [pg/mL] *median (iqr)*	18.9 (38.8)	9.5 (27)	0.367 ^b^
**CPM** *(score 0/1/2/3/4)*	9/7/3/4/1	8/8/4/4/0	0.872 ^c^
**IL1a-polymorphism** *(cc/ct/tt)*	13/12/1	14/7/3	0.320 ^c^
**IL1ß-polymorphism** *(cc/ct/tt)*	16/7/3	17/5/2	0.785 ^c^
**IL1RA-polymorphism** *(cc/ct/tt)*	2/9/15	1/9/14	0.866 ^c^
tnf-α**-polymorphism** *(aa/ga/gg)*	1/4/21	0/4/20	0.623 ^c^

**Table 2:** Bacterial assessment in the different groups

	Test	Control	*p*-value
**Bacterial analysis**	**[n]** *(no/low/high/very high)*	**[n]** *(no/low/high/very high)*	**p-value** ^c^
*A. actinomycetemcomitans* *P. gingivalis* *T. forsythensis* *T. denticola* *P. intermedia* *P. micros* *Fusobacteria* spp. *C. rectus* spp *E. nodatum* *E. corrodens* *C. gingivalis/cochracea*	22/1/0/0 8/2/1/12 2/2/9/10 8/5/4/6 16/1/2/4 8/3/5/7 2/1/10/10 11/5/7/0 20/3/0/0 14/3/6/0 19/2/0/2	24/0/0/0 17/2/1/4 18/2/2/2 19/3/1/1 20/1/1/2 15/5/1/3 5/9/6/4 18/2/4/0 24/0/0/0 14/6/2/2 15/6/3/0	0.302 0.065 > 0.001 **0.016** 0.700 0.070 **0.010** 0.152 0.067 0.289 0.059

^a^ - Fisher’s exact test ^b^ – Mann-Whitney-u-test. ^c^ – Pearson’s Chi-square test

*iqr* inter quartile range, *PPD* Peri-implant pocket dept,; *IgA* immunoglobuline A, *MBL* mannose-binding lectine, *aMMP-8* activated metallomatrix proteinase 8, *tnf-α* stimulated tumor necrosis factor α, *IL-1ß* stimulated interleucine 1ß, *CPM* cytokine polymorphism

Bold *p*-values indicate statistically significant inter-group differences

The scores for CPM levels were in the test and control groups were 9/7/3/4/1 and 8/8/4/4/0, respectively, corresponding to the scores of 0–4. The means and interquartile ranges (IQR) of aMMP8 levels in the respective groups were at 28.2 (60.7) and 34 (67.7) ng/ml. No statistically significant inter-group differences were observed for any of the assessed immunological parameters, including the cytokine polymorphism profile, IgA, mannose-binding lectin, tnf-a macrophage stimulation, IgA status, genetic cytokine profile, bacterial load or aMMP-8 (Table 2). Furthermore, when considering tnf-α values exceeding 40 pg/ml or IL-1ß values exceeding 30 pg/ml as a “positive” sensitization reaction [33], no correlation was found with peri-implantitis.

## Discussion

Within the test-control setting of the present study on 48 patients no correlation was observed between clinical peri-implantitis parameters and laboratory parameters related to tissue macrophage sensitization. Likewise, bacterial levels for eleven so-called key pathogens were not associated to any of the laboratory parameters assessed. Consequently, the study hypothesis was rejected. In the test group, peri-implantitis was confirmed by the presence of pockets exceeding 4 mm with bleeding upon gentle probing, while control patients generally exhibited healthy sites without bleeding. Concentrations of IgA, MBL, tnf-α and IL1ß were generally elevated and exceeding the reference levels [34]. The CPM values in both groups were relatively low, with most cases (65%) scoring 0 and 1 out of 4. With aMMP-8 levels around 30 ng/ml in both groups the values were in a range reported for sites with peri-implant mucositis and lower than those that have been previously been reported to be associated with peri-implantitis [35]. These levels were also close to those observed in patients with periodontitis grades b and c [36], thereby underpinning the clinical findings. In this context, the fact that the participants in the present study were recruited from a cohort of patients with a history of periodontitis undergoing periodontal maintenance therapy may explain the generally elevated levels of cytokines and inflammation markers, Even if perfectly controlled as stated in the inclusion criteria, inflammation parameters in patients with a history of periodontitis, thus proving to be susceptible to an enhanced inflammatory reaction might still be altered as compared to patients without previous periodontal issues. Important to state, however, briefv that this refers to both, test and control group.

Published data on polymorphisms in the encoding regions for the involved cytokines present inconsistent findings. In the present study no intergroup differences were observed in terms of altered gene polymorphisms encoding for IL1α, IL1ß or the concentration of these cytokines. A recent cross-sectional study conducted in Portugal corrobated these findings concerning the investigated polymorphisms encoding for both IL-1α and ß in implant patients with and without peri-implantitis [37]. On the other hand, a recent case-control study involving 120 individuals with healthy implants and peri-implantitis found that the polymorphism in the IL-1ß receptor antagonist was more prevalent in the group of peri-implantitis patients (OR 3), although no statistically significant differences were observed in the proportion of altered alleles for IL-1β between the groups.

A recent retrospective study investigating TO2 sensitization demonstrated a significant correlation with an odds ratio of 19 for clinical symptoms of peri-implantitis correlated to positive testing for TiO_2_ sensitization [33]. However, in this study, “positive” was defined as tnf-α values exceeding 40 pg/ml or IL-1ß values exceeding 30 pg/ml. In contrast, the present data analysis did not reveal such a correlation.

The present study has two major limitations. Although a total of 49 patients provided sufficient power to properly test the hypothesis regarding relevant differences in blood parameters between the groups, the power might be insufficient to make a general statement about the degree to which respective cytokines, aMMP-8, MBL or inflammation-related polymorphisms are correlated with peri-implantitis. On this behalf and to address this question conclusively, large-scale studies are needed. More specifically, such studies should ensure not only the proper comparability of test and control groups, standardized and well-calibrated clinical measurements, and adequate statistical power, but they should also include patients without a history of periodontitis and cases with more advanced peri-implantitis. A multi-center approach involving centers from different countries and involving patients with different oral treatment needs may help minimizing the potential risk of bias.

Furthermore it is important to underline, that peri-implantitis is a multi-factorial disease, with biofilm as the primary etiologic factor and a large variety of confirmed and unconfirmed risk factors [38, 39]. Accordingly, the complex and highly individual host response in each specific patient involves multiple immunological and environmental factors. While testing for specific gene polymorphisms may be reductionist on one hand, the present design and study aim are important to shed a light on the potential relevance of this specific factor. In this context the present study design with a balanced distribution of age and sex provides the best preconditions possible to assess another valuable piece of the whole puzzle of the peri-implant foreign body reaction, reflecting the interrelationship between individual immunological host response and the phenomenon of peri-implantitis.

The moderate degree of peri-implant inflammation might be considered another limitation of the present study. In fact, test implants were not of hopeless prognosis, what might have rendered the investigation more sensitive, thus creating a more significant intergroup difference. Nevertheless, the actual inclusion criteria might better reflect everyday clinical reality with peri-implantitis at an early and implants that are still reasonable-to-treat.

Several tested bacterial species exhibited significant differences between the groups. Although comprehensive systematic reviews have failed to identify a specific microbiome associated with peri-implantitis [40, 41], the finding that *T. forsythensis*,* T. denticola* and *Fusobacterium* spp. were more frequently in the peri-implantitis group aligns with reports of a higher relative abundance and broader diversity of the flora in peri-implantitis sites [41].

Laboratory assessments of MMP-8, the specifically encoding gene areas and finally the bacterial assessment are – strictly speaking - neither clinical nor parameters directly related to macrophage sensitization. However, these data were collected for two reasons: Firstly, to allow for a more comprehensive illustration of the host, the peri-implant tissues and the composition of the peri-implant biofilm. Secondly, the respective laboratory provides these tests and suggest investigating these parameters likewise, when the blood samples sent for analysis. Within the present study, however, these parameters failed to provide clinically relevant information.

Taken together, with a missing correlation of clinical and laboratory parameters, the data from the present study do not suggest that the respective immunologic analysis provides benefits in terms of a prognostic value for the assessment of the risk for peri-implantitis.

## Conclusion

In the present case-control study the parameters related to TiO_2_-sensitization consistently demonstrated no association with clinical symptoms of peri-implantitis. Within the limitations of this study, these tests do not appear to hold predictive large-scale studies should be conducted to investigate whether testing tissue macrophage sensitization can aid in assessing the individual risk for peri-implantitis.

## Data Availability

No datasets were generated or analysed during the current study.
