# Correction to: Comparison of clinical parameters of peri-implantitis and parameters related to tissue macrophage sensitization on TiO_2_

**DOI:** 10.1007/s00784-024-05948-0

**Published:** 2024-09-20

**Authors:** Philipp Sahrmann, Jens Tartsch, Patrick R. Schmidlin

**Affiliations:** 1 Department of Periodontology, Endodontology and Cariology, University Centre for Dental Medicine, University of Basel, Basel, Switzerland; 2 Private Practice, Kilchberg, Switzerland; 3 Clinic of Conservative and Preventive Dentistry, Center of Dental Medicine, University of Zurich, Zurich, Switzerland

**Correction to: Clinical Oral Investigations**

10.1007/s00784-024-05880-3

In the paper that was published, the second author was presented as Tarsch. The correct spelling should be Tartsch.

The original article has been corrected.

